# The value of cardiac magnetic resonance in the prediction of left ventricular function improvement following acute myocarditis

**DOI:** 10.1186/1532-429X-17-S1-P186

**Published:** 2015-02-03

**Authors:** P Stefan Biesbroek, Esha Kumar, Aernout M Beek, Albert C van Rossum

**Affiliations:** Cardiology, VU University Medical Center, Amsterdam, Netherlands

## Background

Although cardiac magnetic resonance (CMR) has good diagnostic performance in myocarditis, an endomyocardial biopsy (EMB) is still required when treatment against the underlying etiology is considered. Many patients however have a good prognosis following myocarditis and will therefore not benefit from an EMB. The ability of CMR to predict outcomes in these patients may therefore guide selective EMB.

## Methods

All patients admitted with an acute coronary syndrome (ACS)-like presentation of myocarditis, with confirmed diagnosis at baseline according to standard CMR criteria and follow-up examination available, were retrospectively identified in our database. CMR images were analyzed to determine left ventricular (LV) dimensions, LV mass and total amount of late gadolinium enhancement. LGE was quantified using signal intensity >5 SD above normal myocardium and was expressed as percentage of LV mass. Improvement of LV function (LVF) was defined as in increase in ejection fraction (EF) of ≥5%.

## Results

Fifteen consecutive patients were included in the present study. Follow-up CMR was performed at a mean of 282 ± 187 days after admission. At baseline, mean EF was 51 ± 7 % and was impaired in 11 patients (47 ± 4 %) . EF improved in 8 of these 11 patients (73%). Total indexed myocardial mass (TMM) and the extent of LGE at baseline did not differ between patients that improved and those that did not (52.9±12.4 vs. 56.9±21.2, p=0.697; 8.2±6.3 vs. 9.2±6.8, p=0.821). At follow-up, the extent of LGE decreased in patients with and patients without LV improvement (-3.0±5.0 vs. -5.0±4.9, p=0.570). However, TMM decreased in patients that showed EF improvement, and not in patients without improvement (-10.8±7.9 vs. 1.9±4.1, p=0.029).

## Conclusions

The majority of patients with an ACS-like presentation of myocarditis have a good prognosis. The improvement of LVF was accompanied by a decrease in myocardial mass. This decrease in myocardial mass likely reflects the resolution of acute inflammatory changes in acute myocarditis.

## Funding

None.

